# Molecular characterization of cell decay in inflammation and topological assignment of released cfDNA for integrative laboratory and radiological outcome assessment

**DOI:** 10.3389/fcimb.2025.1720862

**Published:** 2026-01-07

**Authors:** Catharina Gerhards, Stefanie Nittka, Volker Ast, Osman Evliyaoglu, Maren Hedtke, Jürgen Hench, Marlis Gerigk, Jörg Krebs, Andreas Teufel, Matthias F. Froelich, Andreas Fischer, Stefan O. Schoenberg, Michael Neumaier, Alexander Hertel

**Affiliations:** 1 Institute for Clinical Chemistry, Medical Faculty Mannheim of the University of Heidelberg, Mannheim, Germany; 2 Department of Pathology, Universitätsspital Basel, Basel, Switzerland; 3 Institute of Medical Microbiology and Hygiene, Medical Faculty of Mannheim, University of Heidelberg, Mannheim, Germany; 4 Department of Anesthesiology and Critical Care Medicine, Faculty of Medicine, University Hospital Mannheim, University of Heidelberg, Mannheim, Germany; 5 Division of Hepatology, Department of Medicine II, Medical Faculty Mannheim, Heidelberg University, Mannheim, Germany; 6 Division of Clinical Bioinformatics, Department of Medicine II, Medical Faculty Mannheim, Heidelberg University, Mannheim, Germany; 7 Department of Radiology and Nuclear Medicine, University Medical Center Mannheim, Medical Faculty Mannheim of the University of Heidelberg, Mannheim, Germany

**Keywords:** ARDS diagnostics and outcome, cell-free nucleic acid, COVID-19, DNA-integrity, epigenetics, integrative medicine, intensive care units, radiomics

## Abstract

**Background:**

Integrative biomarkers could aid in the efficient triage of vulnerable patients with systemic infectious diseases. Thus, we investigated cfDNA integrity, cfDNA epigenetics, and radiomics for their potential as prognostic biomarkers for clinical courses in ARDS and systemic involvement.

**Methods:**

The cfDNA integrity index was established using automated gel electrophoresis (TapeStation) based on the release of cfDNA induced by apoptosis and necrosis in HEK293 and Jurkat cells (discovery cohort). This method was then used to evaluate cfDNA fragmentation patterns in blood samples from participants with COVID-19 or other acute diseases (validation cohort). In this analysis, various cfDNA size intervals (50-130 bp, 130-270 bp, 270-450 bp, 450-640 bp, 640-800 bp) were considered, and both the classic DNA integrity index (247/50-800 bp) and an adjusted index (450/50-800 bp) were assessed for their clinical potential in respiratory diseases. Infinium Methylation 850K assay was utilized for topological assignment of secondary organ damage via epigenetic analysis of cfDNA via deconvolution. In total, 122 samples, including cell culture and patient samples, were analysed.

**Results:**

Participants with ARDS exhibited higher cfDNA concentrations with fragment sizes above 247 bp (p = 0.016). Increased cfDNA fragment sizes were observed in association with ICU admission (p = 0.009) and mortality (p = 0.017). The predominant origin of cfDNA was hematopoietic cells. An elevated epigenetic hepatocyte signal showed a strong correlation with GGT levels (r = 0.79). Hepatocyte-derived cfDNA anticipated later ALT elevation (p = 0.008). ECMO was correlated with selected radiomics parameters (r = -0.84).

**Conclusion:**

Implementing the cfDNA integrity index on an automated gel electrophoresis demonstrated promising results in predicting clinical outcomes like ARDS occurrence and mortality. Therefore, integrating laboratory and imaging resources could enhance the allocation of optimal care and may identify secondary systemic complications, especially liver involvement, as our results suggest reduced lead-time for detecting liver injury.

## 1 Background

Exploring strategies to support the triage of vulnerable patients in infectious diseases is of importance, as recently demonstrated by the high hospital occupancy rates during the COVID-19 pandemic, as well as by the influenza seasons recurring for decades (Bezek et al., 2020; Laurie and Rockman, 2021; Meille et al., 2023). In these scenarios, rapid and accurate diagnostic assessment at the time of first presentation is crucial to determine whether patients should be admitted to the hospital or can safely be managed in an outpatient setting. This initial decision is particularly important in times of overloaded healthcare systems, where effective triage and optimal resource allocation can substantially influence the clinical outcome of each individual (Bezek et al., 2020; Rigby et al., 2022). Therefore, integration of laboratory and imaging resources supporting clinical decisions in respiratory infectious disease with systemic complications like sepsis is of considerable clinical importance.

Acute respiratory distress syndrome (ARDS) and sepsis represent interdependent disease entities that can arise vice versa, as ARDS may trigger a systemic inflammatory response resembling sepsis. However, sepsis may lead to the development of ARDS through endothelial and inflammatory mechanisms (Bos and Ware, 2022; Xu et al., 2024), thus integrative biomarkers for early identification are needed. As state-of-the-art in suspected pneumonia that may result in ARDS, chest computed tomography (CT) imaging plays a crucial role in the diagnosis and assessing the severity of the disease (Garin et al., 2019). Moreover, radiomics parameters and radiological scores, such as the RSNA (Radiological Society of North America) score, have proven valuable in evaluating disease burden (Delli Pizzi et al., 2021; Rocha et al., 2022). These tools facilitate a detailed analysis of lung involvement, allowing for a nuanced understanding of disease progression and helping to stratify patients based on their risk profiles. As previously published, integrating radiomics features with other biomarkers, such as cell-free DNA (cfDNA) concentration, a systemic biomarker of cell decay, could enhance the precision of disease severity assessment, leading to more informed clinical decision-making (Gerhards et al., 2023). This is because cfDNA is released into the bloodstream as a consequence of tissue and immune-cell injury, and can therefore be quantified in plasma as a systemic marker of ongoing cell decay. These diagnostic frameworks established during the COVID-19 pandemic might also be applied in other acute infectious diseases and therefore may have a broader applicability than initially assumed. Thus, we examined a comparative cohort of participants with acute medical conditions, both infectious and non-infectious, for integrative diagnostic and prognostic evaluation.

Beyond features that could encourage initial triage of patients admitted to the hospital, identifying sensitive biomarkers for early detection of secondary complications is comparably crucial for adequate therapeutic intervention. In this context, molecular genetic methods could be employed due to their sensitivity and the wide range of methodological possibilities they offer (Danielle et al., 2024). One promising biomarker is cfDNA, presenting a sensitive surrogate marker for secondary complications, such as for example organ failure (Hovhannisyan et al., 2023). Existing literature describes the DNA integrity score as a quantitative measure reflecting differences in cfDNA fragmentation patterns that arise from distinct modes of cell death. During apoptosis, endonuclease-mediated cleavage leads to highly regular nucleosomal fragments, whereas necrosis results in more heterogeneous and higher molecular weight DNA fragments. The integrity score therefore captures the relative proportion of longer cfDNA fragments and has been established primarily in oncological liquid biopsy research, where it supports the distinction between malignant and benign conditions. This methodological concept may also apply to acute inflammatory and infectious diseases, where different cell death pathways contribute to circulating cfDNA profiles (Ravaioli, 2019; Kumar et al., 2022). However, it is conceivable that necrotic cell death could lead to a more severe clinical course, whereas apoptotic tissue damage might result in earlier and more successful convalescence towards full recovery. Correlating cfDNA findings with chest CT imaging, as part of an integrative approach in respiratory diseases, may have the potential to enhance diagnostic validity. Moreover, discrepant chest imaging and laboratory findings in terms of cfDNA quantification could prompt further targeted investigation. These insights into secondary complications can be inferred not only from cfDNA fragment size but also from epigenetic patterns. Through topological assignment of released cfDNA, this approach could detect organ failure more sensitively than routine laboratory parameters, enabling earlier and more targeted therapeutic interventions in patients with inflammatory diseases.

**Figure 1:**
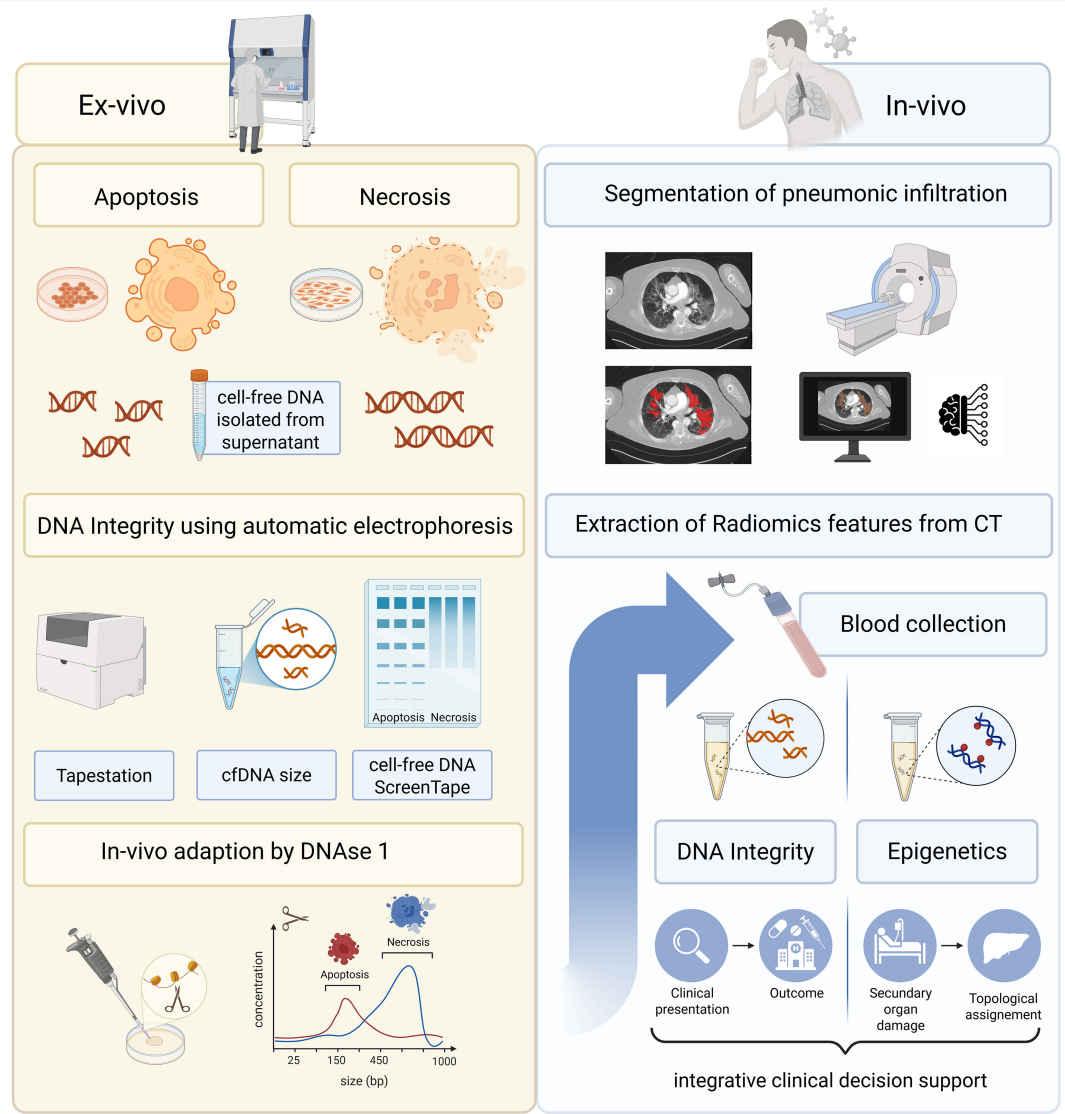
Study design and workflow. First, the methodology was validated through cell culture experiments to distinguish apoptotic and necrotic cell death by use of automatic electrophoresis (TapeStation). Subsequently, clinical samples are analysed to investigate correlations with clinical parameters, imaging findings and epigenetic patterns aiming to identify predictive markers for disease progression. Created in BioRender. Gerhards, C. (2025) https://BioRender.com/i31j626. Color should be used in print.

In this setting, simple and rapid-to-implement procedures need to be assessed due to their applicability in acute situations. Therefore, in our study, the DNA integrity index was measured using an automatic gel electrophoresis method (TapeStation). First, this procedure needed to be verified through cell culture experiments, inducing apoptotic and necrotic cell death to assess the discriminatory potential. The two distinct cell culture lines thus served as a discovery cohort, while the patient cohort provided the clinical validation of the method. Discrepant results to participants’ values were examined in light of physiological differences of human and cell culture conditions to approximate patient blood samples. Finally, the existence of clinical correlations has been investigated. Hence, this study pursues various objectives, including methodological and clinical aspects. More precisely, our study aimed to investigate the applicability of automatic gel electrophoresis for measuring the DNA integrity score (i). Upon methodology establishment, we investigated whether cfDNA is released in an apoptotic or necrotic manner in respiratory infectious diseases (ii), whether the integrity index can serve as an outcome predictor (iii), and if there is a correlation with imaging findings (iv). Furthermore, the potential of epigenetic patterns for early identification of systemic involvement leading to secondary organ damage was evaluated (v). This was achieved through analyses of cfDNA from peripheral blood, in which the proportional contribution of tissue-specific patterns allows inferences on the tissue-of-origin of circulating cfDNA and potentially enables topological assignment of secondary organ involvement (see **Figure 1**).

## 2 Materials and methods

### 2.1 Participant recruitment and sampling timing

Adults aged ≥ 18 years with available chest CT imaging, a previously confirmed Severe Acute Respiratory Syndrome Coronavirus 2 (SARS-CoV-2) infection verified by quantitative polymerase chain reaction (qPCR), and an early blood draw obtained within the first days following the positive qPCR result (sampling time: median 4 days), were enrolled in the LAGGO (Laboratory Assessment of Ground Glass Opacities) study at the University Medical Center Mannheim, Germany (Delli Pizzi et al., 2021). Additionally, a control group consisting of nine non-COVID-19 patients who required intensive care treatment was included in the study. Given the ethical concerns during the initial surge of the SARS-CoV-2 pandemic, obtaining informed consent was deemed inappropriate for patients requiring intensive care. Consequently, participant enrollment was conducted retrospectively post-treatment. The study protocol received approval from the Institutional Review Board (2020-541) and adhered to the principles outlined in the Declaration of Helsinki. These criteria were equally applied to a non-COVID-19 ARDS control cohort from an ongoing study. In this context, the non-COVID-19 control group was augmented with patients with ARDS. This study received approval from the ethics committee (04_0249_S10). Each participant (n = 62) provided informed written consent.

### 2.2 Data collection and analysis

#### 2.2.1 Plasma separation and cfDNA isolation

Ethylene diamine tetraacetic acid (EDTA) anticoagulated whole blood samples obtained via peripheral venipuncture were first centrifuged at 1600× g for 10 minutes at 20°C within 4 hours of blood draw. The resulting supernatant, EDTA-plasma, was then transferred to a fresh 15 ml tube and centrifuged again at 3000× g for 10 minutes. Samples with visible hemolysis upon optical inspection were excluded from further analysis. The final plasma was stored at -80°C until cfDNA isolation. CfDNA extraction was performed using the Qiagen QIAmp Circulating Nucleic Acid Kit (Qiagen, Hilden, Germany) following the manufacturer’s protocol without any modifications. The maximum possible plasma volume from each patient sample (ranging between 0.4 and 1.5 ml) was processed for cfDNA isolation. The quantification of cfDNA was performed using an automated gel electrophoresis (TapeStation, Agilent Technologies, Santa Clara, CA, USA) system with the cfDNA Tape (Agilent Technologies, Santa Clara, CA, USA), and cfDNA concentration was adjusted according to the input volume and reported as ng per ml of plasma. CfDNA was bisulfite-converted and hybridized to Illumina Infinium Human Methylation BeadChips (EPIC v1.0/v2.0, 850K) following the manufacturer’s protocol. Bisulfite-conversion and microarray processing were conducted by Life & Brain GmbH (Bonn, Germany). The resulting IDAT files were converted into beta values. Topological assignment was performed by deconvolution analysis using the published human cell-type methylation atlas by Moss et al (Moss et al., 2018).

#### 2.2.2 Cell culture

Experiments were conducted using Jurkat cells cultured in RPMI-1640 medium and human embryonic-kidney-293-cells (HEK293, ATCC-ID code CRL-1573), in Dulbecco’s Modified Eagle Medium (DMEM), each supplemented with 10% fetal bovine serum (FBS, FBS Superior, S0615, Biochrom) and penicillin-streptomycin ((1%) PenStrep, P4333, Sigma Aldrich). Apoptosis was induced using actinomycin D (0.2 µM) (A9415-2MG, Sigma Aldrich), and necrosis was induced by sodium azide (5 mM) (4221.1 Carl Roth) in triplicates for 24 hours. Apoptosis experiments using Jurkat cells and necrosis experiments by use of HEK293 cells were replicated with DNase 1 (1/10) (DNase I recombinant, RNase-free solution, 4716728001, Roche). For fluorescence imaging, Propidium Iodide (CN74.1, Carl Roth) and Hoechst Dye (33342 Life Technologies)) were used for death cell and nuclear staining and were visualised using an Olympus IX70 inverted microscope (Evident, Tokyo, Japan). Western blot analysis was conducted to detect apoptosis through caspase-8 and -3 activation (Caspase-8 (1C12) Mouse mAb 9746, Cell Signaling; Caspase-3, 31A1067, NB100-56708, Novus Biologicals, both 1/1000) (n = 60 cell culture samples, including cell pellets for western blots and cell culture supernatant for cfDNA analysis). The cell culture lines served as a methodological discovery cohort.

#### 2.2.3 cfDNA integrity index

The cfDNA integrity index was first established in a controlled cell-culture setting using cell culture supernatant samples of apoptosis- and necrosis experiments induced in HEK293 and in Jurkat cells, employing automated gel electrophoresis to characterize fragmentation patterns. Subsequently, this method was used to determine the cfDNA integrity index in a validation cohort of COVID-19 infected participants and patients suffering from other acute diseases in order to assess its performance and validity in a real-world clinical setting. In this context, we evaluated the clinical utility of examining different fragment-size intervals and assessed the need for adapting the established DNA integrity index. Specifically, we analysed total cfDNA, fragment concentrations within defined size ranges (50-130 bp, 130-270 bp, 270-450 bp, 450-640 bp, 640-800 bp), the conventional integrity index (247 bp/total cfDNA), and an adapted index (450 bp/total cfDNA) to determine their respective diagnostic value.

#### 2.2.4 Chest CT imaging

All participants in this study underwent either native or contrast-enhanced chest CT imaging using SOMATOM Definition AS, SOMATOM Definition Flash, or SOMATOM Definition 64 (Siemens Healthcare GmbH, Erlangen, Germany), and RSNA score was calculated as previously published (Moss et al., 2018). Quantitative analysis was performed using radiomics methods with the MM Radiomics Frontier Prototype 1.2.6 (August 2016, Siemens Healthcare GmbH, Erlangen, Germany) within syngo.via VB60A (May 2021, Siemens Healthcare GmbH, Erlangen, Germany). For radiomics feature extraction, the CT Pneumonia Analysis prototype 2.5.2 (April 2021, Siemens Healthcare GmbH, Erlangen, Germany) and Pyradiomics version 2.1.0 were used.

#### 2.2.5 Statistical analysis

For non-normally distributed continuous variables, the Kruskal-Wallis rank sum test was employed. Normally distributed continuous variables were compared by standard ANOVA test. We reported normally distributed variables using the mean and standard deviation, whereas non-normally distributed variables were described using the median and interquartile range. Outcome Analysis was performed via Point Biserial correlation. Comparisons of categorical variables were performed by Fisher’s exact test. P-values of <0.05 were considered statistically significant. Illustrations were created by using Microsoft PowerPoint (2019), R-statistics software (Version 4.1.3 (2022-03-10) and Biorender.

### 2.3 Timeline and setting

Between May 2020 and September 2021, adults with qPCR confirmed SARS CoV 2 infection were enrolled into this study. Blood sampling was obtained in close temporal proximity to the first positive qPCR result. Sampling was performed either at presentation to the emergency department or during the early inpatient course when SARS-CoV-2 positivity was first documented. All EDTA-anticoagulated whole blood samples were collected at the beginning of the infection, with a median of four days after the positive qPCR. If available in the routine clinical setting, remaining diagnostic material was used for longitudinal cfDNA analysis. This approach was only feasible when blood sampling had already been performed as part of standard patient care, as no additional blood draw was undertaken to avoid any undue burden for the patients. Due to the limited consistency of this data point, it was evaluated only for a subset of participants and is reported exclusively in the supplemental material. It was therefore not included as a focus of the present analysis.

## 3 Results

### 3.1 Distinct differences in cfDNA upon apoptosis and necrosis in cell culture

**Figure 2:**
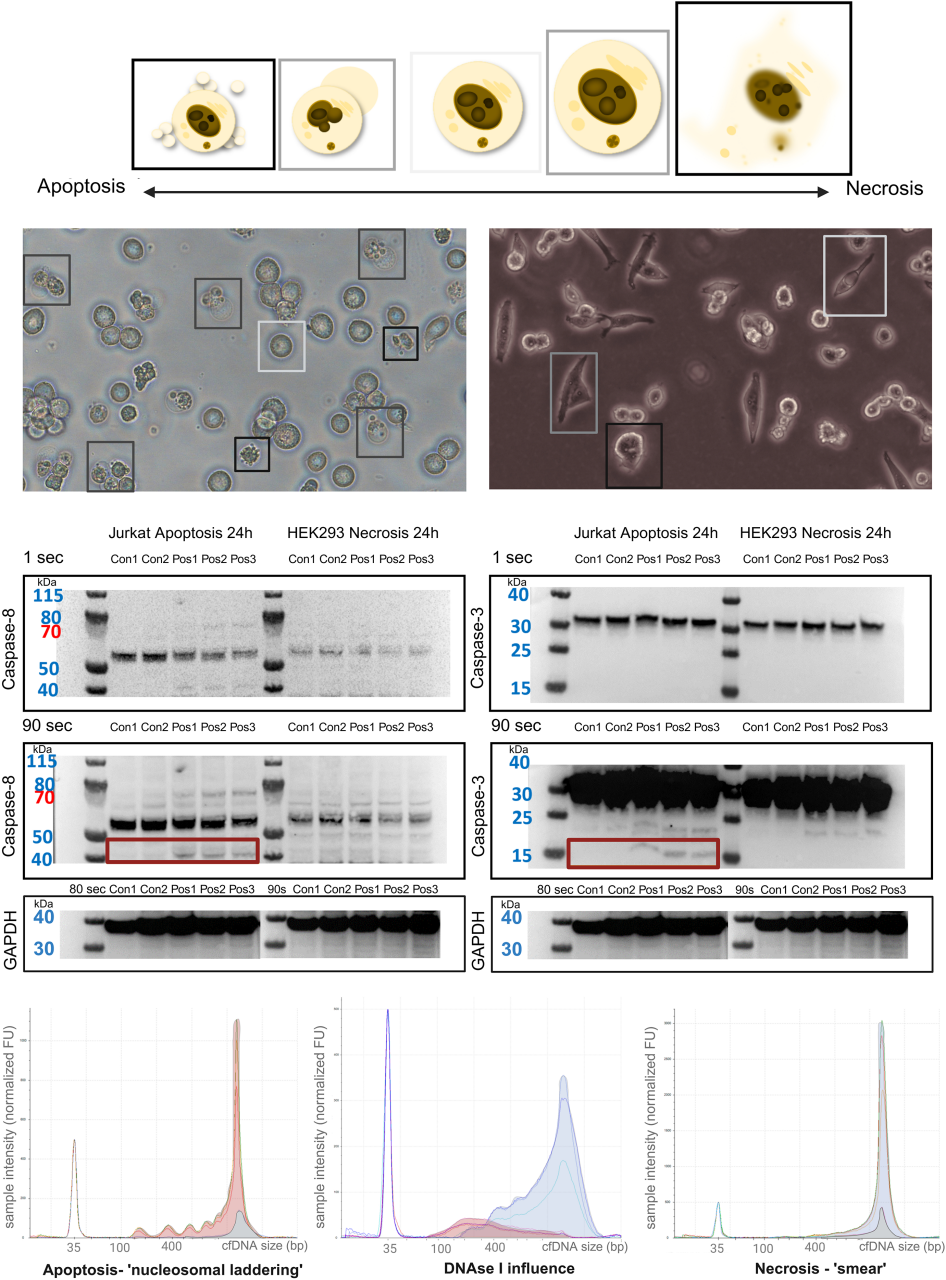
Cell death characteristics in cell culture. Top: Morphological characteristics of apoptotic and necrotic cell death (left: membrane “blebbing” and apoptotic bodies in apoptosis, right: increase in cell volume and membrane rupture in necrosis). Middle: Caspase-8 and -3 activation in apoptosis versus no activation in necrosis in triplicates. Bottom: cfDNA fragment sizes in base pairs (bp) related to sample intensity (normalized FU) (left: nucleosomal laddering in apoptosis, right: “smear” in necrosis, middle: influence of DNase1 on cfDNA released by apoptosis and necrosis, red: apoptosis, blue: necrosis, grey: negative control. Color should be used in print.

Jurkat cells were treated with actinomycin D to induce apoptosis, and HEK293 cells were treated with sodium azide to induce necrosis. This led to typical morphological signs of cell death, such as membrane blebbing and pyknosis in apoptosis, and karyorrhexis in necrosis, observable using native microscopy and fluorescence staining (**Figure 2**; **Supplementary Figure S1**). Caspase-8 activation was demonstrated by detecting the p43/p41 domain with a simultaneous decrease in inactive caspase-8 (approximately 53 kDa) compared to negative controls observed only in the apoptosis experiments. Activated caspase-3 fragments (approximately 15 kDa) are shown in apoptosis experiments only (**Figure 2**).

Moreover, cfDNA was isolated from the cell death experiments’ supernatant. In apoptosis, typical nucleosomal-sized cfDNA laddering was induced in higher concentrations compared to controls (p = 0.013, **Figure 2**, *grey area under the curve*). This pattern arises from the nucleosomal organization of chromatin, where enzymatic cleavage occurs predominantly at linker DNA, producing repeating fragment sizes of ~160-200 bp and thereby generating this typical nucleosomal laddering in apoptotic cell death (Luger, 2006; Jiang and Lo, 2016). However, a continuous distribution (‘smear’) of cfDNA sizes was induced without nucleosomal laddering in necrosis (see **Figure 2**, p = 0.010). The classic DNA integrity index (>247/50-800 bp) averaged 0.78 in the apoptosis experiments and 0.91 in the necrosis experiments (p < 0.001). An overview of the concentrations depending on the base pair size is presented in **Table 1**. These results indicate that apoptosis leads to a proportional increase across all nucleosomal fragment-size intervals, consistent with ordered nucleosomal cleavage. In contrast, necrosis produced higher absolute cfDNA concentrations, particularly in the >270 bp range, reflecting the more heterogeneous, less structured fragmentation pattern typical of membrane-disruptive cell death. With DNase1-enhancement, a DNA integrity index of 0.61 for apoptosis and 0.91 for necrosis was observed (p < 0.001). Adjusting DNA integrity index (>450bp/50-800bp), the DNase1-enhanced apoptosis assays showed a lower index of 0.25 (+/- 0.06) compared to necrosis experiments with an index of 0.61 (+/- 0.04) (p < 0.001) in line with existing literature (Hussein et al., 2019; Shaban et al., 2022). Biologically, this indicates that apoptotic cfDNA, characterized by orderly nucleosomal fragmentation, is more readily degraded into smaller fragments when exposed to DNase I, which reflects *in vivo* nuclease activity. Necrotic cfDNA, in contrast, consists of less structured and more heterogeneous fragments. Furthermore, in DNase1 experiments performed to adapt to *in-vivo*-size of cfDNA, there was a noticeable increase of smaller DNA fragments in the apoptosis experiments compared to the necrosis experiments (see **Figure 2**, *bottom, middle*).

**Table 1:** Results of cell culture experiments.

cfDNA size (bp)	Apoptosis (cfDNA pg/µl)	Control (cfDNA pg/µl)	P-value
50_800 (median [IQR])	258.00 [219.00, 286.00]	30.00 [23.60, 39.95]	0.013
50_130 (median [IQR])	1.80 [1.10, 2.90]	1.43 [0.92, 1.87]	0.518
130_270 (median [IQR])	52.60 [37.90, 61.00]	10.50 [7.99, 11.65]	0.013
270_450 (median [IQR])	68.40 [52.10, 71.50]	5.41 [4.36, 7.80]	0.013
450_640 (median [IQR])	71.40 [66.20, 85.30]	6.49 [5.19, 8.89]	0.013
640_800 (median [IQR])	62.00 [59.60, 74.10]	6.17 [5.13, 9.73]	0.013
247_800 (median [IQR])	200.00 [178.00, 230.00]	18.00 [14.60, 26.30]	0.013

cfDNA size (bp)	Necrosis (cfDNA pg/µl)	Control (cfDNA pg/µl)	P-value
50_800 (median [IQR])	264.00 [264.00, 264.00]	66.20 [60.05, 78.10]	0.010
50_130 (median [IQR])	5.70 [4.20, 6.70]	3.75 [2.36, 4.03]	0.163
130_270 (median [IQR])	20.20 [12.30, 24.00]	2.89 [2.59, 14.60]	0.227
270_450 (median [IQR])	61.40 [52.20, 62.60]	5.80 [4.16, 13.10]	0.012
450_640 (median [IQR])	92.50 [91.20, 96.00]	19.80 [15.05, 26.75]	0.012
640_800 (median [IQR])	89.20 [83.20, 97.80]	27.70 [16.58, 35.80]	0.012
247_800 (median [IQR])	237.00 [228.00, 244.00]	49.70 [44.25, 66.35]	0.012

TapeStation results of apoptosis and necrosis assays without DNase 1 (each experiment n=12).

### 3.2 Results of participants

#### 3.2.1 cfDNA analysis and radiomics

In the overall cohort (n = 62), 77.4% were SARS-CoV-2 positive. The mean age was 66.37 (+/- 15.24) years, with 39% women included. 79% of the participants required intensive care treatment, 14.5% needed extracorporeal membrane oxygenation (ECMO) therapy, and 14.5% showed a lethal outcome. The overall cfDNA concentration (50-800 bp) showed a median value of 107.70 ng/ml [36.23, 264.99]. Among the size-defined sub-intervals (50-130 bp, 130-270 bp, 270-450 bp, 450-640 bp, 640-800 bp), the 130–270 bp range exhibited the highest median concentration, with 82.91 ng/ml [27.91, 212.33]. The mean regular DNA integrity index in our overall cohort was 0.20 (+/-0.06) and a median adjusted score of 0.03 [0.02, 0.04] (**Supplementary Table S2**) with an overall tendency toward apoptotic cell death in the total cohort.

The spectrum of pathogens in non-COVID-19 ARDS ranged from *Citrobacter koseri*, *Aspergillus fumigatus*, to *Mycobacterium tuberculosis* identified from bronchoalveolar lavage. The pathogens in the non-COVID-19 ARDS cases were identified through microbiological analysis of bronchoalveolar lavage samples (culture diagnostics). Higher cfDNA concentrations were observed in participants receiving intensive care treatment compared to those on the general ward (136.50 [45.95, 299.73] versus 35.14 [29.79, 106.91], ICU: n = 49/62, p = 0.012). Moreover, participants suffering from pneumonia and ARDS had significantly higher cfDNA overall concentrations and those having a fragment size above 247bp (pneumonia: p = 0.005, ARDS: p = 0.016) (see **Supplementary Figure S3**). This was not observed when comparing COVID‐19-positive and COVID‐19-negative individuals (p = 0.614). Moreover, significantly more ARDS participants had concentrations >100 ng/ml in the range >247bp (p = 0.030). Furthermore, in deceased individuals, higher concentrations in intervals representing higher fragment sizes were observed (p = 0.017). Furthermore, the regular DNA integrity index showed significantly higher values in non-survivors (p = 0.018). With regard to the RSNA score and the DNA integrity index, there was no significant correlation and only a discrete negative relationship (Spearman = -0.17, p= 0.226) as illustrated in **Supplementary Figure S4**. Further biserial correlation analysis revealed several notable relationships between clinical parameters and outcomes. (**Supplementary Figure S4**). ECMO treatment demonstrated a strong negative correlation with the radiomics texture feature *original_glcm_ClusterShade*, which reflects intensity asymmetry and heterogeneity within affected lung regions (r = -0.84) as illustrated in **Supplementary Figure S4**. A moderate positive correlation was observed between the RSNA score and both ICU admission (r = 0.39) and ARDS development (r = 0.41, p=0.029, see **Supplementary Figure S4**). We identified a significant association between radiomics-derived features and clinical outcomes. The radiomics shape feature original_shape_MajorAxisLength, representing the longitudinal extent of pneumonic involvement, correlated strongly with ICU admission (r = 0.53, p = 0.003), while ‘original_glcm_ClusterShade’ (r = -0.43, p=0.020) exhibited a negative correlation with ARDS severity (see **Supplementary Figure S4**).

#### 3.2.2 Epigenetic profiling of cfDNA to determine cellular source of origin

**Figure 3:**
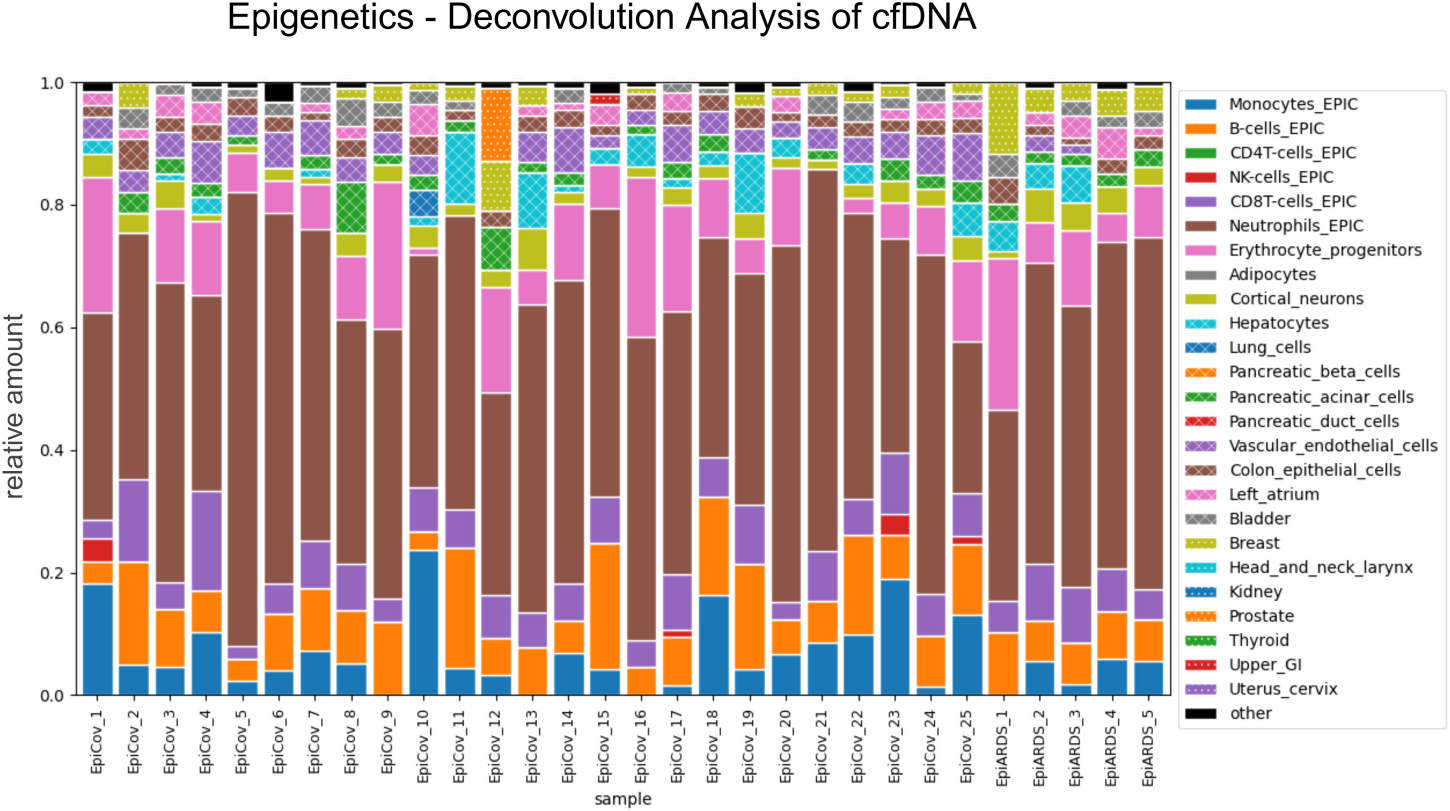
Deconvolution analysis of epigenetics. Epigenetics of COVID-19 and ARDS participants and topological assignment via deconvolution analysis by Moss et al. Hematopoietic cells are the most common origin of cfDNA release. Further secondary organ damage is assigned via images legend. The contribution of the releasing cells is expressed as a fraction of the total. Color should be used in print.

Profiles of cfDNA with Illumina Infinium Methylation Array were performed to determine which cell types preferentially release cfDNA into the blood. In our overall cohort, cfDNA was mainly released from hematopoietic cells (predominantly by neutrophil granulocytes (46% +/- 11%, n=30) and erythrocyte precursor cells (9% +/- 7%) (**Figure 3**). The subsequent most common epigenetic signature was assigned to vascular endothelial (4%) with significant higher proportion in SARS-CoV-2 infected participants (p = 0.001), followed by colon epithelium (3%), cortical neurons (3%), pancreatic acinar cells (2%), left atrium (2%), breast (2%) and bladder (2%). In addition, one patient showed a clear prostate signature (12%), one participant exhibited a high breast signature (12%), and another one showed a lung signature (4%). The former had a first-diagnosed metastatic prostate carcinoma, the patient with the highest breast signal showed radiological evidence of breast carcinoma, and the single patient with lung signal was diagnosed with granulomatosis and polyangiitis.

In addition to the descriptive assessment of cfDNA tissue contributions in predominantly respiratory infected participants, we performed a correlation analysis with routine laboratory parameters. This analysis served both as a reference to an established ground truth and as an approach to identify organs that are particularly well captured through cfDNA profiling.

**Figure 4:**
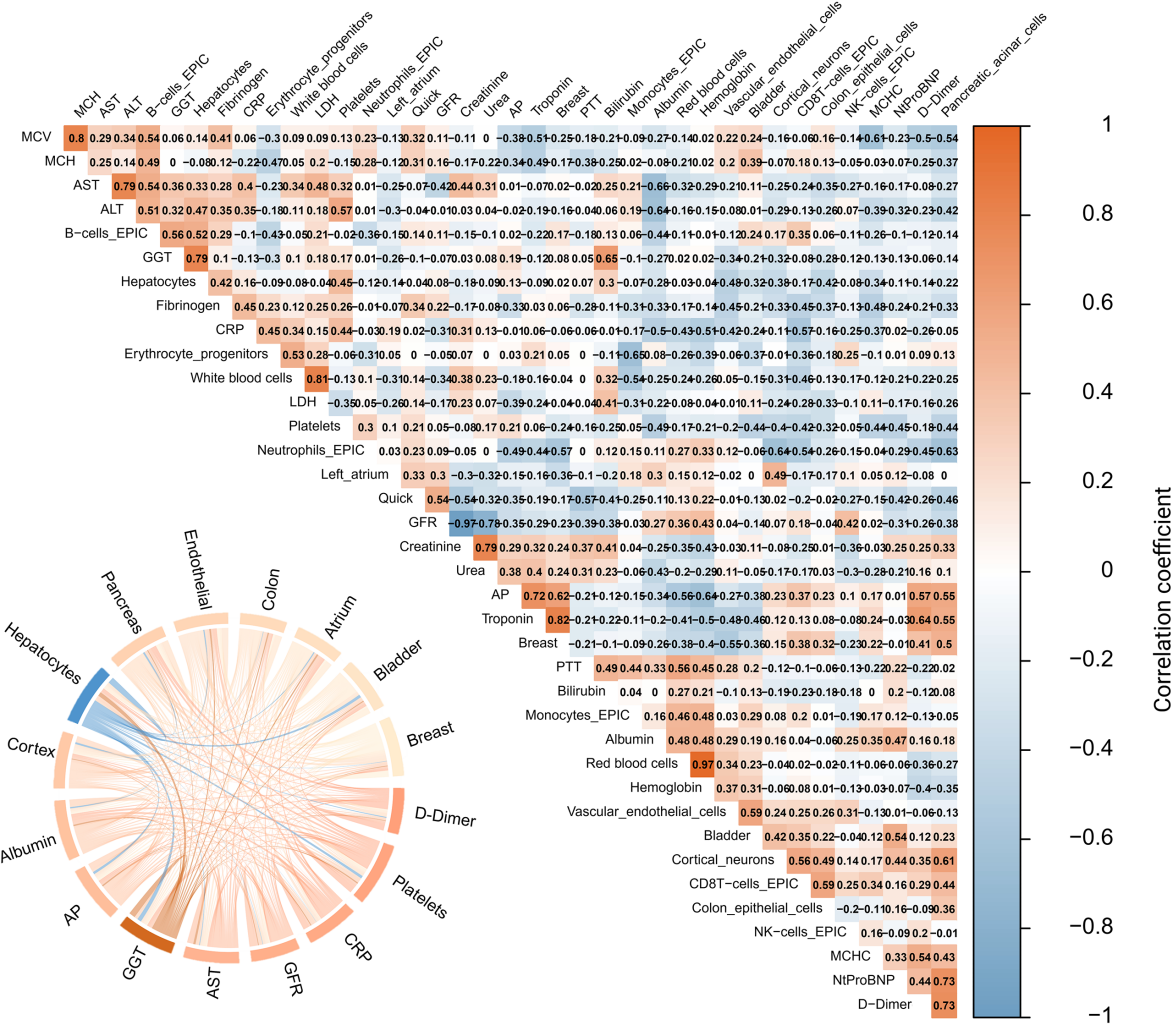
Correlation of epigenetics and routine laboratory. Right: correlation between laboratory parameters illustrated by a correlation plot with respective correlation values. *Red: high positive correlation. Blue: high negative correlation.* Left: illustration of highest correction between epigenetics and routine laboratory via a cord plot. Color should be used in print.

Notably, the fraction in percent of hepatocyte-derived cfDNA, inferred from methylation-based tissue-of-origin deconvolution, showed a strong positive correlation with serum gamma-glutamyltransferase (GGT) concentration (r = 0.79, **Figure 4**, **S5**). From a diagnostic perspective, we were further interested in evaluating the potential added value of cfDNA compared with conventional liver biomarkers such as aminotransferases or GGT. In accordance with previous publications reporting hepatocyte-derived cfDNA fractions of approximately 1% in healthy individuals (Moss et al., 2018), we defined two thresholds to classify pathological hepatocyte signals: Classification A (> 1%) and Classification B (≥ 3%). These two cut-offs were used to stratify participants into groups with borderline versus pronounced elevated hepatocyte-derived cfDNA fractions for comparison of liver enzyme levels and evaluation of lead-time reduction via epigenetic analysis (Moss et al., 2018). In this context, Classification A, characterized by a higher proportional contribution of a hepatocyte-derived epigenetic cfDNA signature exceeding 1% of total cfDNA, exhibited significantly higher alanine aminotransferase (ALT) levels compared to those below the threshold (29.00 [19.00, 43.00] versus 44.00 [32.00, 83.00], p = 0.034). This effect was even more pronounced in the comparison of ALT levels of blood samples collected seven days later than the cfDNA samples (p = 0.008). Notably, median ALT values at the time of cfDNA sampling were still within the normal range, indicating that the epigenetic hepatocyte signal may anticipate the subsequent rise in ALT.

According to classification A (> 1%), GGT levels showed a slight trend toward a significant difference over the course of time (p = 0.087). In classification B, which was defined by an even higher proportion of 3%, significantly higher GGT (84.00 [36.25, 156.50] versus 256.00 [154.00, 535.50], p= 0.008, seven days later: p = 0.046) and AP levels (67.00 [55.25, 98.75] versus 233.00 [136.50, 573.25], p=0.001, seven days later: p = 0.006) were observed. This was investigated to determine whether different types of liver injury (intra- vs. post-hepatic) might be associated with distinct ranges of the epigenetic signal, and to assess whether this analysis could provide a diagnostic advantage compared with routine laboratory testing. Since the detection of a hepatocyte-derived epigenetic signal together with concomitantly elevated aminotransferase levels at the time of sampling could serve as a proof of principle, its diagnostic utility would still need to be questioned in the absence of a potential lead-time reduction.

## 4 Discussion

In this study, we investigated the potential of integrative diagnostics for clinical support in inflammatory lung diseases with systemic involvement, incorporating radiomics and cfDNA analysis. Initially, we established the DNA integrity score using an automatic gel electrophoresis to introduce a rapid and easy-to-implement method and analysed associations with the clinical outcome. Additionally, we explored the potential of epigenetic motifs for detecting secondary organ manifestations during septic complications as a further diagnostic tool in an integrative framework.

In the cell culture experiments, we could detect typical changes in apoptosis, including pyknosis, membrane blebbing, apoptotic bodies, and nucleosomal laddering (Elmore, 2007; Galluzzi et al., 2018) and activation of caspase-3 and -8 (Beroske et al., 2021). CfDNA analysis clearly demonstrated the effective application of the automatic gel electrophoresis (TapeStation) as a screening method for cfDNA integrity assessment to distinguish between apoptosis and necrosis. However, discrepancies were observed between cell culture results and those from patient samples, indicating that a successful translation of the results required adaptation to the *in vivo* situation. While apoptosis and necrosis produced distinct cfDNA fragmentation patterns under controlled conditions, patient samples exhibited fragments around 200 bp. These discrepancies likely reflect the complex *in vivo* situation, where multiple cell death pathways and nuclease activities overlap, leading to partial degradation and mixed cfDNA size profiles. Previous studies have demonstrated that DNase I has a substantial *in-vivo* impact on the overall cfDNA fragment size (Serpas et al., 2019; Han et al., 2020). To approximate the *in-vivo* situation within our artificial cell-culture system, we replicated the apoptosis and necrosis experiments with the addition of DNase I. This resulted in a clear shift toward smaller cfDNA fragments in the apoptosis-DNase I conditions, whereas the necrosis-DNase I experiments continued to show predominantly larger fragments. Serpas et al. demonstrated an influence of DNase I and IL3 on the cfDNA size in mouse experiments (Serpas et al., 2019; Han et al., 2020). Therefore, we extended our cell culture conditions to include DNase I and applied the integrity score to these results. Moreover, we evaluated an adapted DNA integrity index, which yielded results for apoptosis and necrosis comparable to the literature (Schwarzenbach et al., 2011; Fawzy et al., 2016; Kumar et al., 2022; Shaban et al., 2022). However, this adaptation was only necessary in cell culture conditions and not for patient samples. This might be because of the artificial aspect of cell culture. Nevertheless, we observed a clear development of cfDNA into smaller fragments during apoptosis, whereas this shift was absent in the necrosis experiments. This supports the findings of previous publications, as it confirms that cfDNA maintains a larger size in necrosis (Schwarzenbach et al., 2011; Ungerer et al., 2021). Thereby, we were able to demonstrate the applicability of the automatic gel electrophoresis for assessing the DNA integrity score. In the literature, real-time PCR was used for this purpose, employing qPCR-Alu115 (representing total cfDNA) and qPCR-Alu247 (corresponding to high molecular weight DNA) (Hao et al., 2014; Vizza et al., 2018). One significant benefit of implementing the DNA integrity score via automatic gel electrophoresis is the ability to selectively evaluate DNA fragments above 247 bp while maintaining the potential to focus on other cfDNA size ranges. This approach shows promise in correlating with the occurrence of ARDS and thus might have a clinical impact as a predictive marker.

Regarding our patient cohort, the cfDNA integrity score presented values indicative of cfDNA release in an apoptotic manner. This might be due to the predominant release of cfDNA by leucocytes as revealed by our epigenetic screening analysis. In terms of cfDNA integrity score, we identified a significant association with a fatal outcome (p = 0.018). Furthermore, in intervals with higher cfDNA fragment size, deceased patients showed higher concentrations, highlighting the potential utility as an outcome biomarker. Previous work has shown that focusing on higher cfDNA fragment sizes and the DNA integrity index has diagnostic value in oncology, where it supports the discrimination between benign and malignant conditions (Petit et al., 2019; Yu et al., 2019; Kumar et al., 2022; Tagawa and Aoki, 2023). However, the relevance of cfDNA quantity and fragmentation patterns extends well beyond cancer. In infectious diseases and sepsis, cfDNA concentrations are substantially elevated and closely correlate with disease severity and mortality. Combining cfDNA levels or integrity indices with established inflammatory markers improves early diagnosis and risk stratification (Jing et al., 2022; Dadam et al., 2024). Similarly, in acute cardiac injury, such as myocardial infarction, cfDNA is rapidly released from apoptotic and necrotic cardiomyocytes. Several studies have demonstrated high diagnostic accuracy for cfDNA-based markers in detecting myocardial injury, with cfDNA peaks reflecting infarct size and predicting adverse outcomes (Tan et al., 2023; Rafiei et al., 2025). Collectively, these findings demonstrate that cfDNA concentration and fragmentation patterns are not limited to oncological applications but represent broadly applicable biomarkers of tissue damage and systemic inflammation across diverse clinical settings. In our study, the implementation of specific interval differentiation revealed a novel aspect of DNA integrity within the clinical context of respiratory diseases with systemic involvement. Thus, evaluating cfDNA fragment sizes exceeding 247 bp may aid clinical decision-making and triage for patients suffering from ARDS with high potential of septic complications, as our results revealed associations with ICU demand and lethal outcome.

From an integrative perspective, radiomics parameters also demonstrated relevance in discriminating patient outcomes. Particularly, radiomics showed potential in the prediction of ICU admission and ARDS, with a high correlation between ,*original_shape_MajorAxisLength*’ and ICU and ARDS. Since ,*original_shape_MajorAxisLength’* is a shape feature that quantitatively reflects the spatial extent of segmented infiltrates in lung imaging, its strong correlation with disease severity provides a logical correspondence. This association suggests that larger infiltrate dimensions in imaging are indicative of more severe disease manifestations, reinforcing the clinical relevance of radiomics-based severity assessment. Conversely, *original_glcm_ClusterShade* exhibited a negative correlation with ARDS, indicating its potential role in differentiating disease states. Since ,*Cluster Shade’* quantifies the asymmetry and uniformity of the gray-level co-occurrence matrix, a lower value indicates a more homogeneous texture with less pronounced asymmetry. This finding could reflect the loss of structural heterogeneity in lung infiltrates as ARDS progresses, where diffuse alveolar damage leads to more uniform and confluent opacities in imaging. The reduction in ,*Cluster Shade’* may thus be indicative of more severe lung involvement, aligning with the pathophysiological changes seen in ARDS. This suggests that texture-based radiomics features could play a role in differentiating disease severity and may contribute to improved imaging-based assessment of ARDS progression.

In addition to the evaluation of the primarily affected organ for assessing the progression of ARDS, the identification of systemic involvement constitutes a further critical aspect in determining the overall patient outcome. Therefore, in cases of discrepant chest CT-radiomics and systemic laboratory findings, progressive organ involvement should be considered, which may warrant additional epigenetic analyses for a more comprehensive assessment. Thus, the potential of epigenetic cfDNA analyses as a marker of secondary organ damage was evaluated, and a particular benefit was observed in cases with liver involvement, particularly in cases of post-hepatic and more superficial liver injury. The delayed but increasing significance of ALT elevation observed in routine laboratory analyses seven days later may indicate potential for lead time reduction, especially considering that median ALT values were still within the normal range at the time of cfDNA sampling. In the context of progressive hepatic damage leading to more than 3% of epigenetic hepatocyte signal, there appears to be a specific association with post-hepatic processes, as evidenced by a pronounced increase in GGT and AP levels. The utility of epigenetic signatures of cfDNA has been shown in existing literature in the context of Hepatocellular Carcinoma (HCC) and liver cirrhosis (Buzova et al., 2022; Zhou et al., 2022; Bai et al., 2024; Pollastri et al., 2025). Moreover, Lehmann-Werman et al. reported that liver-derived cfDNA may be significantly elevated in sepsis patients with hepatic involvement, which substantiates our findings (Lehmann-Werman et al., 2018). While their study employed a liver-region-specific ddPCR approach, we utilized the Illumina Infinium Methylation Assay in combination with deconvolution analysis based on the reference database by Moss et al (Moss et al., 2018). This method, applied to patients with infectious diseases, might represent a broader and more comprehensive screening marker instead of only focusing on one type of organ damage.

It has been previously described that ARDS and sepsis are closely interconnected and often mutually reinforcing conditions, whereby ARDS can progress into a systemic inflammatory state with secondary multiorgan involvement consistent with sepsis (Bos and Ware, 2022). However, sepsis itself can precipitate the development of ARDS through systemic inflammation. In this context, endothelial injury has been identified to play a crucial role in the pathogenesis of septic ARDS (Bos and Ware, 2022; Xu et al., 2024). In our patient cohort, epigenetic profiling revealed a significantly higher proportion of cfDNA originating from vascular endothelium in patients with COVID-19 compared to controls. This finding may be interpreted in the context of septic ARDS as well as COVID-19–related endothelial activation, which is characterized by the release of proinflammatory cytokines, activation of the complement system, and NETosis (Valencia et al., 2024). In this regard, an association between NETosis-triggered endothelial injury and septic ARDS has been shown (Valencia et al., 2024), providing a plausible explanation for the pronounced vascular signal observed in our cohort. Consequently, this epigenetic cfDNA signature may represent a promising biomarker for the early identification of septic ARDS in the future. Taken together, these findings highlight the potential clinical value of combining cfDNA-based integrity and epigenetic analyses with radiomics to support early triage decisions and to sensitively detect secondary organ involvement at an earlier stage than routine laboratory parameters. Such an integrative approach could meaningfully impact patient management in acute inflammatory diseases, although validation in an independent cohort will be essential before clinical implementation.

Although these findings highlight the potential of the results presented, certain limitations must be considered. First, the cohort size was limited due to high mortality during the recruitment period in the first wave of the COVID-19 pandemic, and a retrospective contact with relatives was not pursued due to ethical considerations. Despite this limitation, we expanded the cohort by including additional participants from an ongoing study involving ARDS patients. This not only increased the sample size, enhancing the validity and robustness of the findings, but also broadened the diversity of the pathogen spectrum examined. In addition, material obtained from routine clinical care was used, which meant that the timing of blood sampling and imaging was not fully synchronized across participants and may therefore have influenced the strength of correlations between cfDNA parameters and radiomic features. This issue was addressed by harmonizing the dataset through the use of the earliest available blood sample, obtained a median of 4 days after the first positive qPCR, and aligning it with the closest corresponding chest CT scan. Nevertheless, we acknowledge that additional, study-specific sampling would have yielded a more consistent dataset. However, this was not feasible for imaging, as study-related radiation exposure would not have been ethically justifiable. In light of these considerations, the chosen approach was implemented and taken into account when evaluating the validity of the data. Moreover, the translation of controlled *in vitro* cell culture findings to the *in vivo* patient setting is inherently limited by complex biological factors, including circulating nucleases and overlapping cell death pathways contributing to cfDNA release. Finally, as a single-center, exploratory study, the generalizability of our findings remains restricted and warrants validation in larger, multicenter cohorts with standardized analytical workflows. Finally, although the onset of the pandemic in 2020 was characterized by high hospitalization rates and lethality, subsequent SARS-CoV-2 variants have been associated with lower clinical severity. As a result, the clinical impact of COVID-19 at the time of sample collection may differ from later phases of the pandemic. This expansion of the dataset to include other infectious and non-infectious diseases represents a valuable extension of the study due to a broader spectrum of application. This is particularly noteworthy because the diagnostic value of assessing larger cfDNA fragments was observed in pneumonia and ARDS, but not in COVID-19. Consequently, our findings extend beyond SARS-CoV-2-related disease.

The relevance of identifying broadly applicable biomarkers is in line with previous research, as Langelier et al. highlighted the utility of diagnostic and prognostic biomarkers in pneumococcal infections (Langelier et al., 2020), while Zhai et al. emphasized the importance of appropriate markers in influenza, known for its recurring infection periods with potentially fatal outcomes (Zhai et al., 2015). Therefore, the translational results of our study offer promising potential for introducing novel integrative biomarkers in patients with suspected pneumonia. Moreover, incidental findings or indications of malignant diseases were observed in two patients. In one case, a metastatic prostate carcinoma was confirmed during hospitalization (Gerhards et al., 2024). Another patient exhibited radiological evidence suggestive of breast cancer, which was further supported by the epigenetic signature identified in our study data. These observations underscore the broad applicability of the methods presented here. However, the sample size of these two participants remains too small for robust statistical conclusions, highlighting the need for investigation in subsequent studies.

In summary, this study explored novel biomarkers and contributed to the translational application of these cfDNA markers in an integrative setting with lung-CT radiomics for patients with ARDS and secondary systemic involvement. The findings demonstrated the methodological and clinical utility of the DNA integrity score, particularly when analyzing specific fragment size intervals. This emerged as a suitable marker for predicting ARDS occurrence and clinical progression. Its predictive power may be further enhanced by combining it with specific radiomics features and the RSNA score. Regarding detailed topological mapping of secondary organ damage, the use of epigenetic methods to detect liver involvement revealed implications in lead-time reduction. This approach could enable early, targeted imaging-based abdominal investigations, supporting its use as an integrative diagnostic tool for both patient triage during initial evaluation and complication monitoring throughout hospitalization. Thus, combining laboratory and imaging resources to support clinical decision-making may improve the allocation of optimal care and facilitate the early detection of secondary complications.

In conclusion, our study highlights the potential of integrative diagnostics combining cfDNA analysis, epigenetics, and radiomics for outcome assessment in ARDS and systemic inflammatory diseases. The DNA integrity score measured by automated gel electrophoresis reliably distinguished apoptotic from necrotic cfDNA patterns *in vitro* and proved feasible for clinical application. Higher concentrations of cfDNA fragments >247 bp and an elevated integrity index were associated with ARDS, ICU admission, and mortality, suggesting value as early outcome predictors. Moreover, radiomics features, particularly shape- and texture-based parameters, correlated with clinical endpoints, supporting their utility as imaging biomarkers of disease severity. In terms of systemic involvement, epigenetic deconvolution of cfDNA enabled early identification of secondary organ involvement, with indication for hepatocyte-derived cfDNA anticipating later ALT elevation. This integration of laboratory-, imaging-, and epigenetic data may enhance early triage decisions and improve detection of systemic complications in patients with severe respiratory infections. Future studies in larger, multicenter cohorts are needed to validate these findings and further refine integrative diagnostic workflows.

## Data Availability

The original contributions presented in the study are included in the article/**Supplementary Material**. Further inquiries can be directed to the corresponding author.

## Supplementary material

Supplementary Figure 1 Viability staining in apoptosis and necrosis. Top shows Hoechst Dye staining to illustrate the nuclei (left apoptosis, right necrosis). Bottom shows fluorescence imaging using Propidium iodide for identification of cell death (left apoptosis, right necrosis.

Supplementary Figure 2 cfDNA and clinical outcome (Table).

Supplementary Figure 3 Clinical association of cfDNA fragment size. Violin charts presenting cfDNA concentration with higher fragment sizes (above 247 bp) for clinical outcome.

Supplementary Figure 4 Correlation matrix of clinical endpoint and integrative variablesCorrelation between laboratory and radiomics parameters with clinical outcome illustrated by a correlation plot. *Red: high positive correlation. Blue: high negative correlation.*

Supplementary Figure 5 Longitudinal assessment of epigenetics. The longitudinal course was analysed in six patient samples, showing a clear increase in hepatocyte signal over time.
