# Supplementary figures and images for: Molecular characterization of cell decay in inflammation and topological assignment of released cfDNA for integrative laboratory and radiological outcome assessment
Source: Front Cell Infect Microbiol. 2026 Jan 7;15:1720862. doi: 10.3389/fcimb.2025.1720862 (PMC12819667; doi:10.3389/fcimb.2025.1720862)

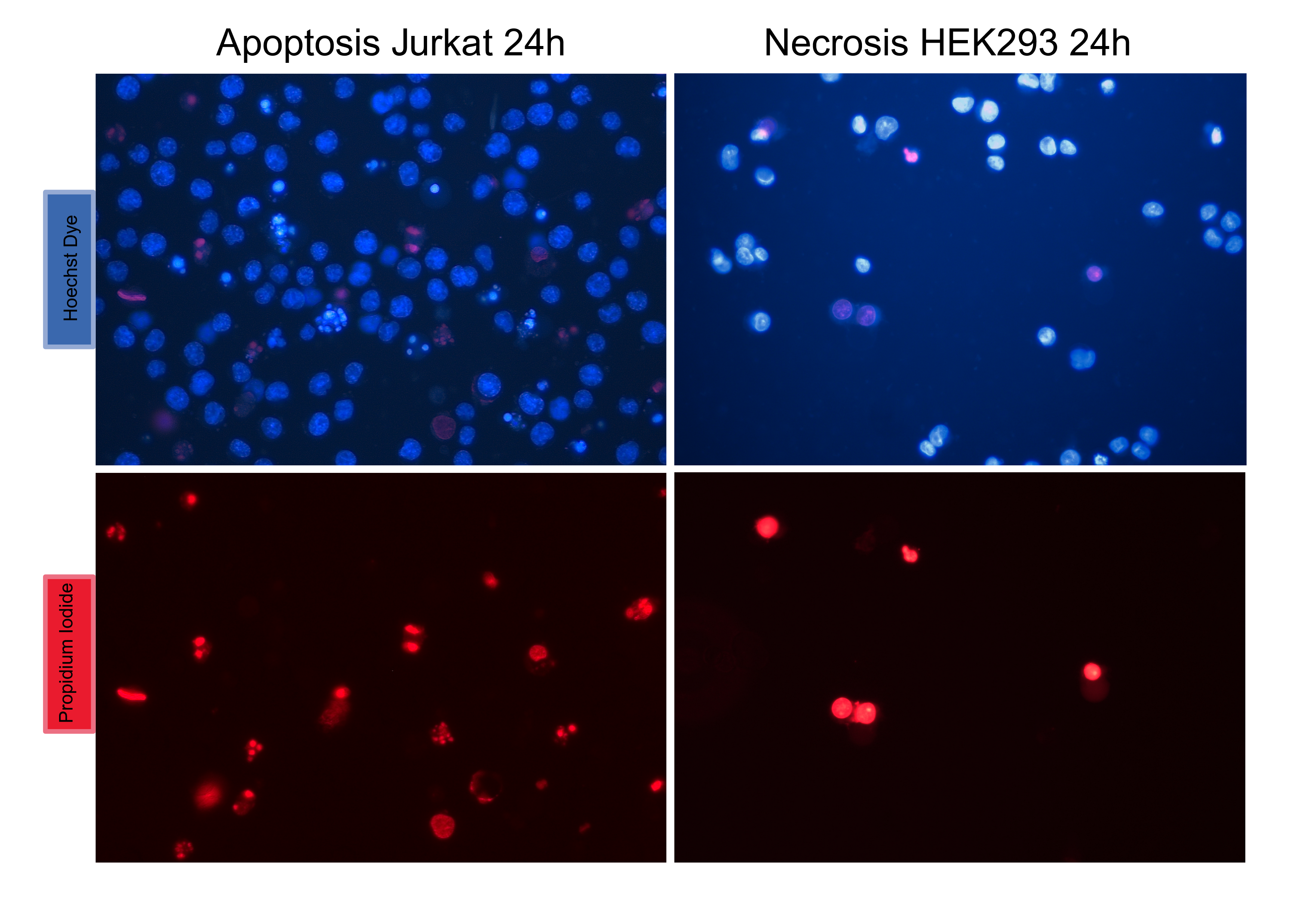

Supplement: Supplementary Figure 1 — Viability staining in apoptosis and necrosis. Top shows Hoechst Dye staining to illustrate the nuclei (left apoptosis, right necrosis). Bottom shows fluorescence imaging using Propidium iodide for identification of cell death (left apoptosis, right necrosis. [file Image1.png]

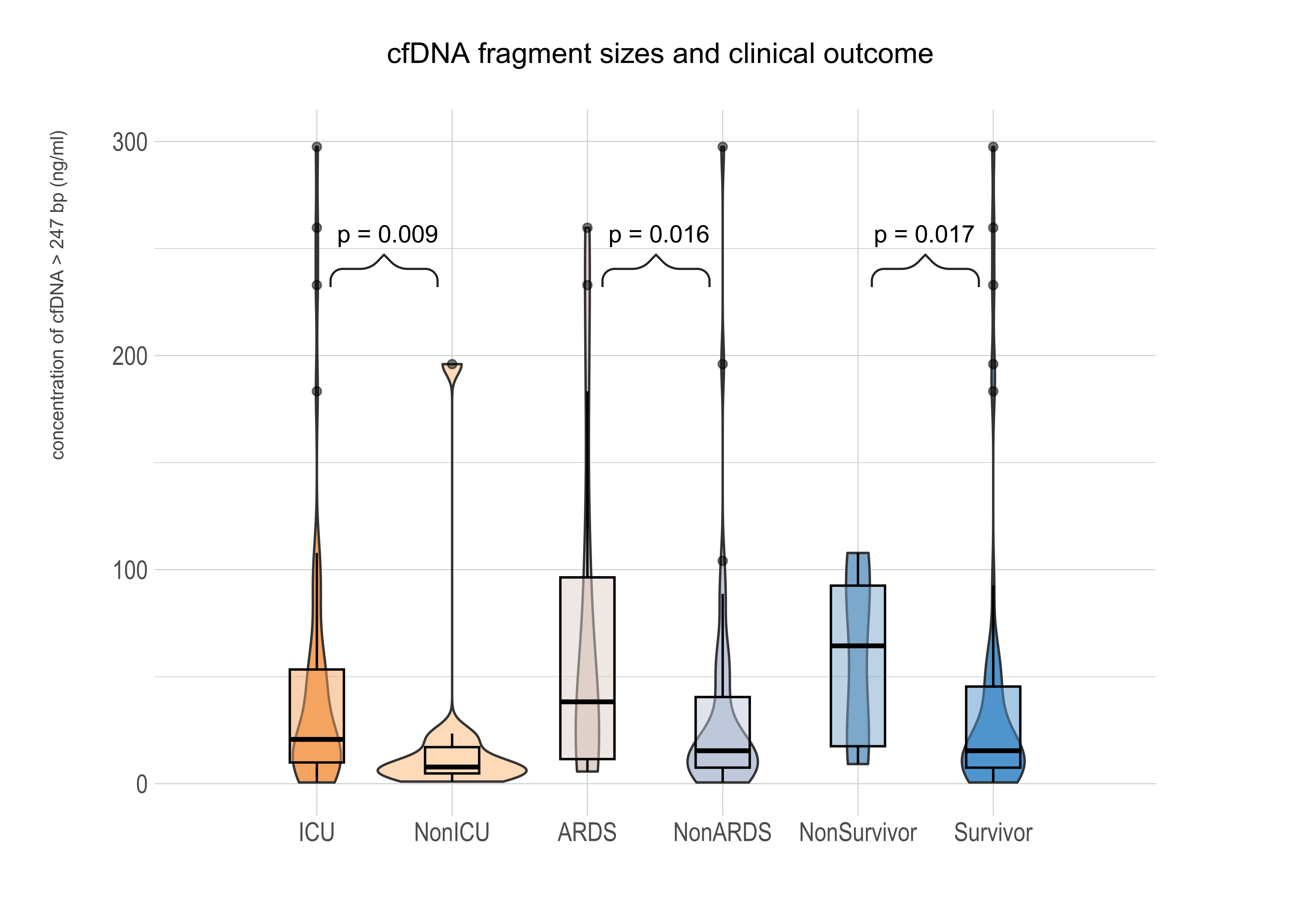

Supplement: Supplementary Figure 2 — cfDNA and clinical outcome (Table). [file Image2.png]

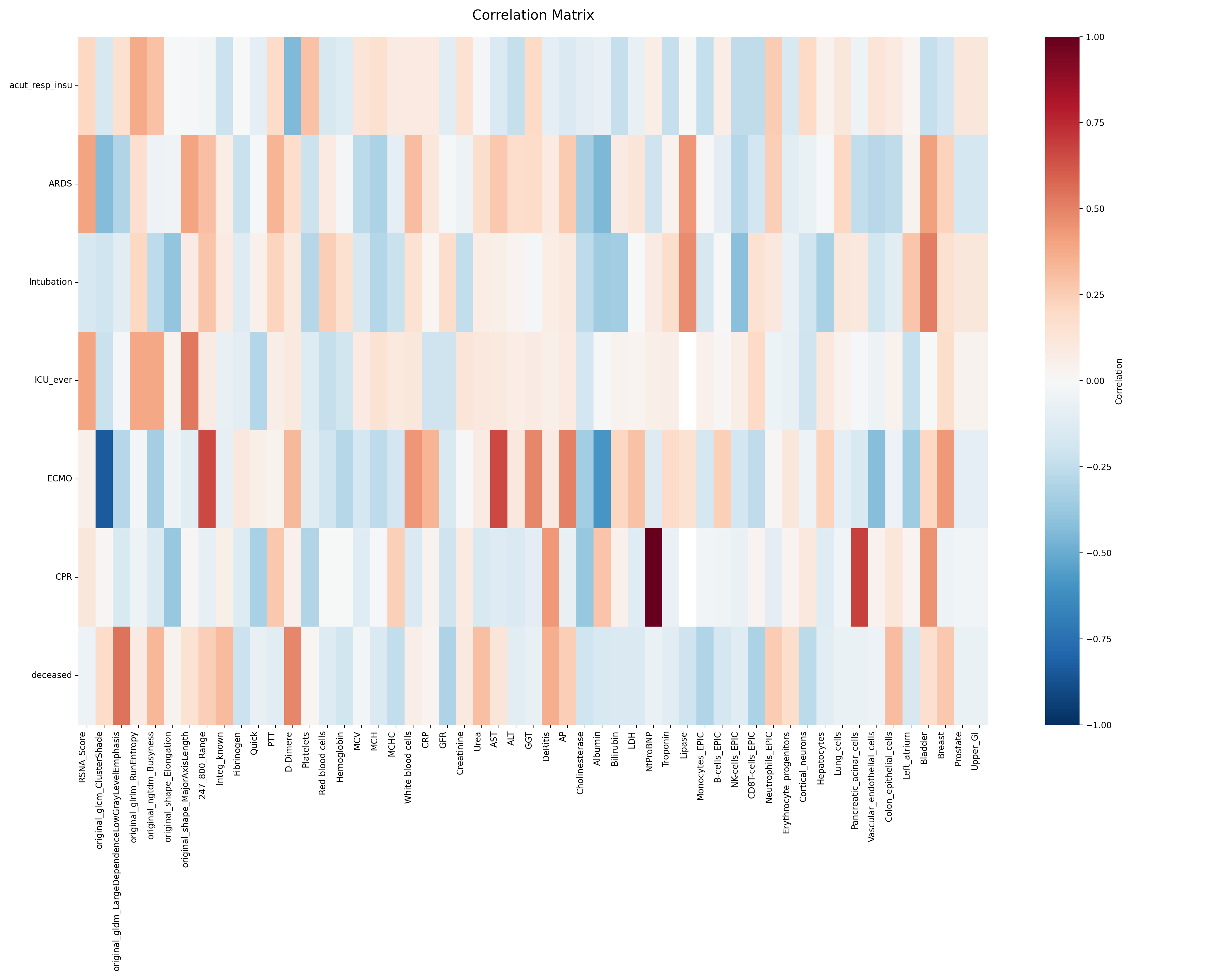

Supplement: Supplementary Figure 3 — Clinical association of cfDNA fragment size. Violin charts presenting cfDNA concentration with higher fragment sizes (above 247 bp) for clinical outcome. [file Image3.png]

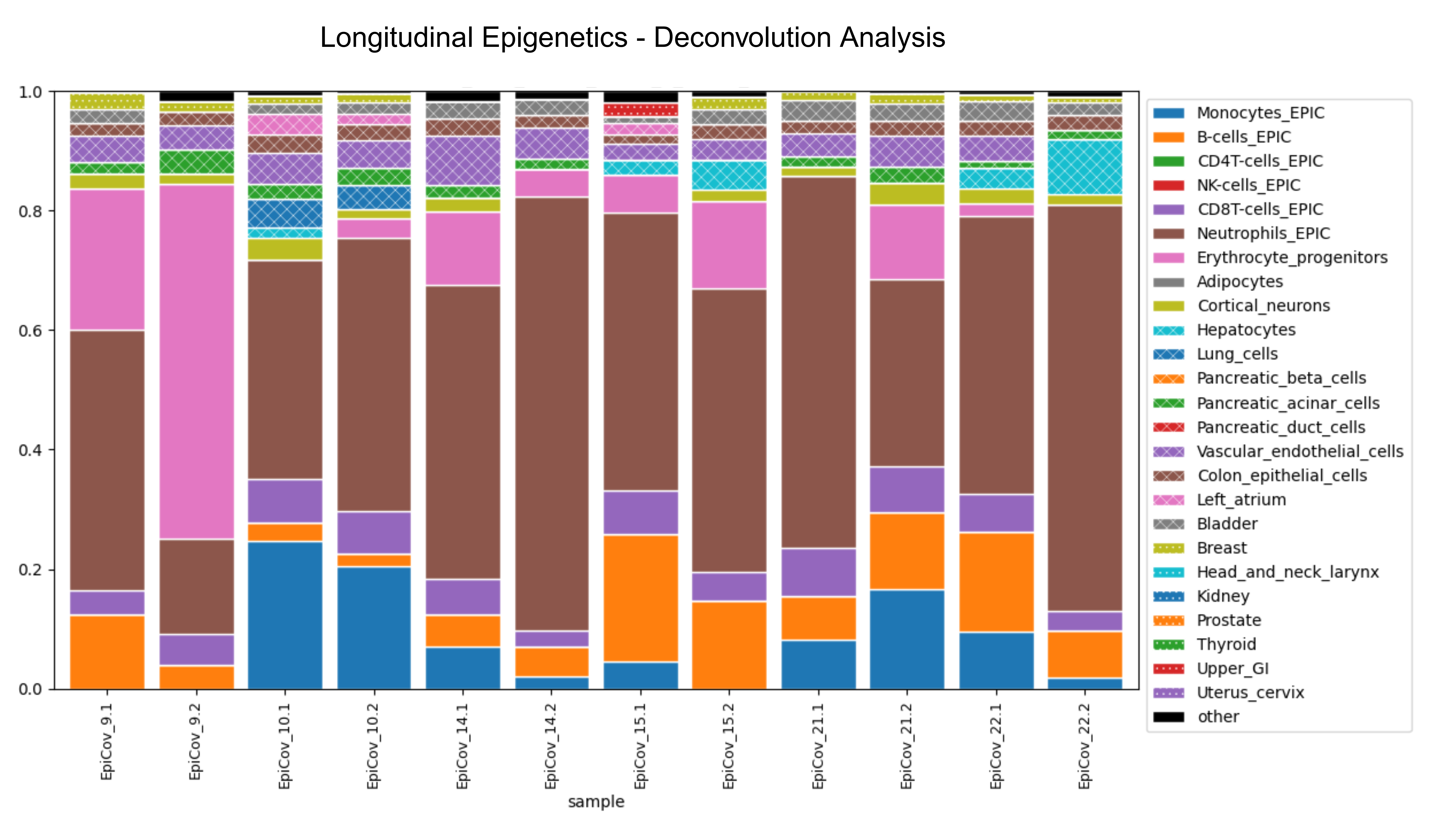

Supplement: Supplementary Figure 4 — Correlation matrix of clinical endpoint and integrative variablesCorrelation between laboratory and radiomics parameters with clinical outcome illustrated by a correlation plot. Red: high positive correlation. Blue: high negative correlation. [file Image4.png]
