# Supplementary material for: Molecular characterization of cell decay in inflammation and topological assignment of released cfDNA for integrative laboratory and radiological outcome assessment
Source: Front Cell Infect Microbiol. 2026 Jan 7;15:1720862. doi: 10.3389/fcimb.2025.1720862 (PMC12819667; doi:10.3389/fcimb.2025.1720862)
Supplement: Supplementary file 5 [file Table1.docx]

**Table 2: cfDNA and clinical outcome**

| **Parameter** | **Non-COVID** | **COVID** | **p-value** |
| --- | --- | --- | --- |
| cfDNA 50-800 bp (median [IQR]) ng/ml | 17.50 [11.75, 24.75] | 11.54 [0.00, 22.26] | 0.066 |
|  | **Non-ICU** | **ICU** |  |
| cfDNA 50-800 bp (median [IQR]) ng/ml | 35.14 [29.79, 106.91] | 136.50 [45.95, 299.73] | 0.012 |
| cfDNA 247-800 pb (median [IQR]) ng/ml | 7.77 [4.76, 17.06] | 22.23 [10.05, 60.51] | 0.009 |
| Integrity Index (mean (SD)) | 0.21 (0.05) | 0.20 (0.06) | 0.623 |
| cfDNA 247-800 bp > 100 ng/ml (%) | 1 (7.7) | 7 (14.3) | 1.000 |
|  | **Non-ARDS** | **ARDS** |  |
| cfDNA 50-800 bp (median [IQR]) ng/ml | 71.10 [34.04, 196.00] | 235.90 [57.48, 432.83] | 0.031 |
| cfDNA 247-800 pb (median [IQR]) ng/ml | 15.40 [7.51, 40.49] | 45.38 [11.58, 107.80] | 0.016 |
| Integrity Index (mean (SD)) | 0.20 (0.06) | 0.21 (0.06) | 0.655 |
| cfDNA 247-800 bp > 100 ng/ml (%) | 3 (6.7) | 5 (29.4) | 0.030 |
|  | **Non-ECMO** | **ECMO** |  |
| cfDNA 50-800 bp (median [IQR]) ng/ml | 99.75 [36.11, 208.25] | 551.25 [57.48, 1470.00] | 0.088 |
| cfDNA 247-800 pb (median [IQR]) ng/ml | 16.61 [8.33, 45.43] | 88.67 [9.41, 232.87] | 0.084 |
| Integrity Index (mean (SD)) | 0.20 (0.06) | 0.18 (0.04) | 0.340 |
| cfDNA 247-800 bp > 100 ng/ml (%) | 4 (7.5) | 4 (44.4) | 0.012 |
|  | **survivor** | **Non-survivor** |  |
| cfDNA 50-800 bp (median [IQR]) ng/ml | 99.75 [35.14, 206.23] | 379.17 [71.10, 551.25] | 0.059 |
| cfDNA 247-800 pb (median [IQR]) ng/ml | 15.40 [7.51, 45.38] | 83.42 [17.80, 104.12] | 0.017 |
| Integrity Index (mean (SD)) | 0.19 (0.05) | 0.25 (0.08) | 0.018 |
| cfDNA 247-800 bp > 100 ng/ml (%) | 5 (9.4) | 3 (33.3) | 0.083 |
